# Analysis of population genetic structure and gene flow in an annual plant before and after a rapid evolutionary response to drought

**DOI:** 10.1093/aobpla/plv026

**Published:** 2015-03-27

**Authors:** Rachel S. Welt, Amy Litt, Steven J. Franks

**Affiliations:** 1 Department of Biological Sciences, Fordham University, Bronx, New York, NY 10458, USA; 2 The New York Botanical Garden, Bronx, New York, NY 10458, USA; 3 Present address: Department of Herpetology, American Museum of Natural History, New York, NY 10024, USA; 4 Present address: Botany and Plant Sciences, UC Riverside, Riverside, CA 92521, USA

**Keywords:** *Brassica rapa*, climate change, drought, gene flow, phenological isolation, population structure, resurrection study

## Abstract

The impact of environmental change on population structure is not well understood. This study aimed to examine the effect of a climate change event on gene flow over space and time in two populations of *Brassica rapa* that evolved more synchronous flowering times over 5 years of drought in southern California. Using plants grown from seeds collected before and after the drought, we estimated genetic parameters within and between populations and across generations. We expected that with greater temporal opportunity to cross-pollinate, due to reduced phenological isolation, these populations would exhibit an increase in gene flow following the drought. We found low but significant *F*_ST_, but no change in *F*_ST_ or Nm across the drought, in contrast to predictions. Bayesian analysis of these data indicates minor differentiation between the two populations but no noticeable change in structure before and after the shift in flowering times. However, we found high and significant levels of *F*_IS_, indicating that inbreeding likely occurred in these populations despite self-incompatibility in *B. rapa*. In this system, we did not find an impact of climate change on gene flow or population structuring. The contribution of gene flow to adaptive evolution may vary by system, however, and is thus an important parameter to consider in further studies of natural responses to environmental change.

## Abstract (precis)

Climate change can have widespread and devastating impacts, but little is known about the effects of this change on the genetics of natural populations. We examined the genetics of two populations of an annual plant (*Brassica rapa*) before and after an extended drought in California. We found limited genetic differences between pre-drought ancestors and post-drought descendants, indicating that the two populations did not become more genetically similar, despite a greater overlap in reproductive timing after the drought. The findings of this study contribute to an understanding of the genetic impacts of climate change, which is critical in optimizing conservation efforts.

## Introduction

Climate change is a major environmental concern and one of the greatest threats to biodiversity (Williams *et al*. 2008; Bellard *et al*. 2012). Climate can influence natural systems from a genetic to an ecosystem level, affecting individuals' fitness and species' survival, and is a major factor in determining community composition and ecosystem function (Bellard *et al*. 2012). The complex relationships between biotic and abiotic factors, combined with the intricate responses of species to a changing climate and other threats to biodiversity, make understanding the current and future impacts of global climate change difficult as well as of great importance (Parmesan and Yohe 2003; Pearson and Dawson 2003; Walther 2010).

Climate change can act as a strong selective pressure in natural populations (Jump and Peñuelas 2005; Gienapp *et al*. 2008; Hoffmann and Sgró 2011). Several recent studies have illustrated the potential for rapid evolution in response to changes in species' surroundings (Huey *et al*. 2000; Stockwell *et al*. 2003; Franks *et al*. 2007). Adaptive responses in both plants and animals frequently involve the evolution of phenological traits (Fitter and Fitter 2002; Peñuelas *et al*. 2002; Menzel *et al*. 2006; Hoffmann *et al*. 2010), which often depend on seasonal cues, such as temperature and/or precipitation conditions (Franks *et al*. 2007; Jarrad *et al*. 2008). Consequently, changes in climate patterns can have major impacts on phenology, and evolutionary changes in phenology may further affect adaptive ability by altering patterns of gene flow between populations (Garant *et al*. 2007; Franks and Weis 2009). For example, in populations that are differentially affected by changes in environmental conditions, phenology (i.e. timing of reproduction) may become more or less synchronized, impacting the opportunity for reproduction across populations.

Generally, gene flow is expected to reduce local adaptation by homogenizing populations found in differing environments, or by spreading detrimental alleles across populations (Ellstrand *et al*. 1989; Holt and Gomulkiewicz 1997). However, gene flow may also serve to introduce potentially adaptive alleles to populations, and increase genetic diversity, which natural selection can act upon to provide an evolutionary response (Ellstrand *et al*. 1989; Räsänen and Hendry 2008; North *et al*. 2011; Sexton *et al*. 2011). An improved understanding of both the effects of climate on evolutionary changes in phenology, and the consequences of these phenological changes for gene flow and local adaptation, will be valuable in predicting how species may respond to changes in climate (Garant *et al*. 2007).

Adaptation can provide a response to changes in climate in some natural populations (Franks *et al*. 2014). Franks *et al*. (2007) found that an extended drought in southern California led to an adaptive response in the flowering times of two populations of *Brassica rapa* (field mustard). The drought, lasting from 2000 to 2004, corresponded to a shift to earlier mean flowering time in both populations, causing an abbreviated growing season and resulting in more synchronous flowering times between the populations following the drought (Franks *et al*. 2007; Franks and Weis 2009).

Seeds were collected from both sites in May 1997 (prior to the drought) and June 2004 (immediately following the drought), at the end of the growing season for both populations in these years. A parent-offspring analysis from a greenhouse study demonstrated an additive genetic basis for this evolutionary change in flowering time (Franks *et al*. 2007). Additionally, further analysis has shown a decrease in phenological isolation of the two sites between 1997 (Isolation Index = 33 %) and 2004 (Isolation Index = 14 %) as a result of the change in flowering time (Franks and Weis 2009).

The current study focuses on the impacts of the change in flowering times on the genetics of these natural populations. We analysed the population genetics of this system before and after the drought to examine genetic structuring and gene flow across this phenological change. While previous studies have used population genetic data to infer historic levels of gene flow and genetic structure, this study is unique in using the ‘resurrection approach’ (Franks *et al*. 2008) of comparing population genetic patterns in ancestors and descendants using stored seeds.

Based on previous analyses of this system (Franks *et al*. 2007; Franks and Weis 2009), we expected to find an increase in gene flow between the populations following the drought, corresponding to decreased phenological, and potentially reproductive, isolation. Given the long-distance (>5 km) pollinator-mediated pollen dispersal found in related species (Devaux *et al*. 2005, 2007), we expected that our sites (3 km apart) would be connected by some degree of gene flow. Regardless, our approach allowed us to determine genetic changes that occurred both within and between populations over time. Changes in population structure would not only elucidate how an adaptive evolutionary change influenced genetic patterns, but may also have implications for the ability of these populations to further evolve in response to continued changes in climate. The results of this study can contribute to the growing understanding of natural responses to climate change events, which will be necessary in aiding in the conservation of species as the environment continues to change.

## Methods

### Study system

*Brassica rapa* is a weedy plant, native to Eurasia but currently distributed worldwide with cultivars and naturalized populations (Suwabe *et al*. 2002). The populations of interest in this study are found in southern California, where *B. rapa* was introduced ∼300 years ago and has become naturalized (Franks *et al*. 2007). In these populations, *B. rapa* is an annual plant, produces perfect flowers and is self-incompatible. Once an individual plant has begun to flower, many new flowers may open each day, each flower lasting ∼3 days, for the remainder of the growing season. In southern California, *B. rapa* germinates in the winter, flowers in the spring and continues to produce flowers until the end of the rainy season (late spring to summer). Flowers are pollinated by many species of vagile insects, such as bees and flies, allowing for the dispersal of pollen across these insects' foraging range (Davis *et al*. 1994; Warwick *et al*. 2003).

**Figure 1.**
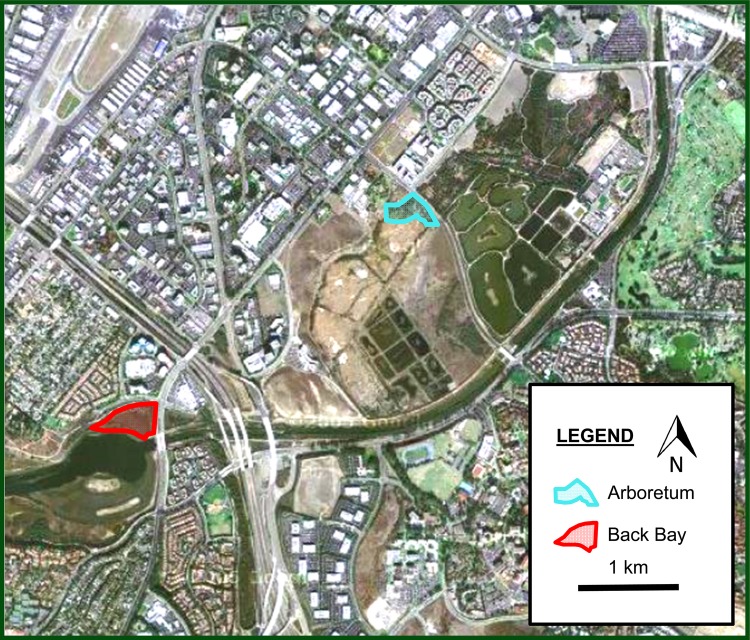
Map of Irvine, California showing the location of the two populations: Arboretum and Back Bay.

The populations were located in upper Newport Harbor Back Bay (BB) and near the San Joaquin Marsh Arboretum (Arb), in Irvine, CA, USA (Fig. 1). The two locations are ∼3 km apart and are characterized by differing soil conditions. Back Bay soil is sandy and dry in comparison to Arb, where the soil has a higher water table and clay content, aiding in moisture retention (Franke *et al*. 2006). These two populations also differed in their flowering time ranges, with BB flowering earlier than Arb prior to the drought (Franks *et al*. 2007). Both populations comprised hundreds to many thousands of individuals both before and after the drought.

### Samples

Seeds collected from individuals in the Arb and BB populations in 1997 and 2004 were stored at 4 °C. Over 10 000 seeds were collected from individuals across both populations to obtain representative samples of the gene pools. Seeds from this stock were grown for tissue collection on light carts in the Franks laboratory at Fordham University. Plants were watered daily and fertilized weekly yielding 259 total samples (56 Arb 1997, 39 BB 1997, 89 Arb 2004 and 75 BB 2004) (Table 1).

**Table 1.** Genetic diversity characteristics by population and year: population size (*N*), per cent polymorphic loci (P), number of PA, number of alleles per locus (A), expected heterozygosity (*H*_e_), observed heterozygosity (*H*_o_) and inbreeding coefficient (*F*_IS_). Standard error is provided in parentheses. An asterisk indicates significance from zero at *P* < 0.05.

Population	Year	*N*	P (%)	PA	A	*H*_e_	*H*_o_	*F*_IS_
BB	1997	39	90	0	3.700 (0.633)	0.517 (0.071)	0.332 (0.057)	0.369*
BB	2004	75	100	8	5.000 (0.715)	0.503 (0.066)	0.349 (0.065)	0.309*
Arb	1997	56	100	4	4.200 (0.629)	0.490 (0.065)	0.332 (0.065)	0.330*
Arb	2004	89	90	3	4.100 (0.737)	0.453 (0.068)	0.301 (0.066)	0.340*

### DNA extraction

Leaf tissue was collected from individuals following the development of true leaves. Approximately 50 mg of tissue per individual was removed, dried and stored in silica gel until extraction. Dried tissue was homogenized using Qiagen's TissueLyser system (Qiagen, Valencia, CA, USA). DNA was extracted using a Qiagen DNeasy plant kit following manufacturer's DNeasy 96 Protocol. This extraction process yielded 200 µL of eluted DNA, which was stored at −20 °C.

### Microsatellite analysis

Ten microsatellite loci were chosen based on repeatable amplification success and high degree of polymorphism: BN12A (Szewc-McFadden *et al*. 1996), Na10-A08, Na10-D09, Na10-G10, Ni4-A03, Ol10-D08, Ra2-E04, Ra2-E12 (Lowe *et al*. 2002, 2004), BRMS-040 and BRMS-037 (Suwabe *et al*. 2002). PCR was carried out in 12.5 µL reactions using TC-5000 thermal cyclers (Techne, Bibby Scientific Ltd, Staffordshire, UK) to isolate and amplify microsatellite loci. These reactions included 0.5 µL of unlabelled forward and reverse primers, 0.5 µL of template DNA, RNase-free water and AmpliTaq Gold 360 Master Mix (Life Technologies Inc., Grand Island, NY, USA). Cycling protocol began with an initial denaturation at 95 °C for 5 min, followed by 30 cycles of amplification at 95 °C for 30 s, optimized annealing temperatures **[see Supporting Information]** for 30 s, and 72 °C for 30 s. This concluded with a final extension at 72 °C for 7 min.

A second PCR was run to incorporate a fluorescent label (WellRED Dye D2, D3 or D4) (Sigma-Aldrich Co., St. Louis, MO, USA) using 0.5 µL reverse primer and 0.5 µL fluorescently labelled primer (M13F(-20) [D2], M13F(-40) [D3] or M13R [D4]) corresponding to the tag sequence added to the 5′ end of the initial forward primer. This was included in a reaction with 0.5 µL of 1 : 100 diluted PCR product from the first PCR, along with RNase-free water and AmpliTaq Gold 360 Master Mix. This second PCR followed the same cycling protocol as the first. PCR products were run on 1 % agarose gel to confirm the presence of target microsatellite fragments. Fragment length was then analysed with a Beckman Coulter CEQ 8800 using Genetic Analysis System v.9.0 (Beckman Coulter, Fullerton, CA, USA). Samples were run with 0.5 µL of Beckman Coulter 400 bp Size Standard and PCR product at volumes according to their fluorescent label: 2.5 µL of M13F(-20), 2.5 µL of M13F(-40) and 1.5 µL of M13R. Alleles were scored using GENEMARKER v.1.90 (SoftGenetics, State College, PA, USA).

### Statistical analysis

GENALEX v.6.41 (Peakall and Smouse 2006) was used to determine measures of microsatellite diversity, including number of alleles (A), expected heterozygosity (*H*_e_), observed heterozygosity (*H*_o_) and inbreeding coefficient (*F*_IS_) for each locus. ARLEQUIN v.3.5.1.3 (Excoffier and Lischer 2010) was then used to calculate Hardy–Weinberg Equilibrium (HWE) and to test for linkage disequilibrium (LD) across all loci. Departure from HWE was determined for each locus using the Markov chain method with a length of 1.0 × 10^5^ steps (Guo and Thompson 1992). Linkage disequilibrium was determined using an Expectation Maximization algorithm with 1.0 × 10^4^ permutations (Lewontin and Kojima 1960; Slatkin 1994; Slatkin and Excoffier 1996). The frequency of null alleles for a locus was estimated using an Expectation Maximization algorithm (Dempster *et al*. 1977) in GENEPOP v 4.0.10 (Raymond and Rousset 1995; Rousset 2008). These parameters describe the loci and can indicate the influence that each has in defining measures of structure in these populations.

GENALEX was also used to determine measures of allelic diversity within these populations including polymorphism (P) across all loci, the number of private alleles (PA), A, *H*_e_ and *H*_o_. *F*_IS_ was calculated using ARLEQUIN for each population. *F*-statistics, such as *F*_IS_ (inbreeding) and *F*_ST_ (fixation), are used to describe the fixation of alleles for individuals in a population, and for populations in a metapopulation, respectively (Wright 1951; Weir and Cockerham 1984). Several analogues of *F*_ST_ have been developed to account for the mutational characteristics of microsatellites (Slatkin 1995; Michalakis and Excoffier 1996; Hedrick 2005; Jost 2008). In addition to *F*_ST_, we also calculated *ϕ*_ST_ (Michalakis and Excoffier 1996), which takes into account the step-wise mutation model believed to be characteristic of many microsatellites. Because *F*_ST_ and gene flow are inversely related (Wright 1951), we used *F*_ST_ to test the hypothesis that gene flow increased following the evolutionary shift to greater flowering synchrony between the populations.

Pairwise comparisons of *F*_ST_ and *ϕ*_ST_, as well as AMOVA were performed in GENALEX. To determine whether levels of fixation and gene flow changed across this period, we compared *F*_ST_ values across these years. Significance was established using 95 % confidence intervals (CI) as determined through 1000 bootstrapping replicates to estimate *F*_ST_ using GDA v.1.1 (Lewis and Zaykin 2002).

Bayesian estimates of genetic clustering probabilistically assigns individuals to populations defined by allele frequencies at multiple loci, and were determined using STRUCTURE v.2.3.2.1 (Pritchard *et al*. 2000) for 5.0 × 10^4^ burn-in repetitions and 1.0 × 10^6^ MCMC simulations at four iterations. This allowed for an estimate of the number of genetic units, K, following Evanno *et al*. (2005) using STRUCTURE HARVESTER web v.0.6.92 (Earl and vonHoldt 2012). Outputs of four iterations at *K* = 3 were run in CLUMPP v.1.1.2 (Jakobsson and Rosenberg 2007) to align these clustering results, and were then visualized using DISTRUCT v.1.1 (Rosenberg 2004).

Data were reanalysed using only loci in HWE in each population **[see Supporting Information]**, as the analyses assume that these microsatellites are acting as neutral markers and a deviation from HWE may be an indication of an effect of selection. The same procedures were used to estimate *F*_IS_ within each population, *F*_ST_ and *ϕ*_ST_ between Arb and BB in 1997 and 2004 and to compare *F*_ST_ between the 1997 and 2004 generations. Results for these analyses are noted along with the results of analyses run with all 10 loci.

## Results

### Allelic characteristics

Across the populations, the loci analysed showed a mean polymorphism of 95 % per population (Table 1). Between two and nine alleles were found per locus (mean = 5.8 alleles per locus) **[see Supporting Information]** and between zero and eight PA were found in each population (Table 1). Of the 38 polymorphic loci (for 10 loci across four populations), 26 exhibited departure from HWE (*P* < 0.05). Out of 180 total locus pairs, 22 pairs across all populations showed LD. However, these locus pairs were not linked in each population. The estimated frequency of null alleles ranged from <1.0 × 10^−4^ to 0.3558 for all loci. The loci in HWE were found to have a lower frequency of null alleles. Therefore, supplementary analyses performed using only loci in HWE, also minimized the influence of allelic dropout.

### Intra-population characteristics

We found high genetic diversity, and some changes in the number of PA, but no significant change in overall genetic diversity over time **[see Supporting Information]**. In the BB population, the number of PA increased from zero to eight between the sampling years (Table 1). The mean number of alleles per locus estimated for these samples did not change significantly (3.7 ± 0.633 (±SE) in 1997 to 5.0 ± 0.715 in 2004) (Table 1) **[see Supporting Information]**. However, in Arb, the number of PA decreased from four to three, with no significant change in the number of alleles per locus (4.2 ± 0.629 in 1997 and 4.1 ± 0.737 in 2004) (Table 1) **[see Supporting Information]**.

All populations showed fewer than expected heterozygotes and high levels of inbreeding (*F*_IS_) (Table 1). *F*_IS_ was significantly greater than zero for all populations and generations (Table 1). Levels of *F*_IS_ did not change significantly over time **[see Supporting Information]**. Measures of *F*_IS_ using only loci in HWE showed lower levels of inbreeding (between 0.065 for Arb97 and 0.085 for BB04) **[see Supporting Information]** than the analyses performed using all 10 loci (Table 1), which is expected as random mating is an assumption of HWE.

### Genetic structure and gene flow

We estimated fixation between all pairs of populations (BB 97, BB 04, Arb 97 and Arb 04) as *F*_ST_ and *ϕ*_ST_. Fixation between populations was low but significant for all pairs except BB 97 and BB 04. *F*_ST_ ranged from 0.027 (between Arb 97 and Arb 04) to 0.039 (BB 97 and Arb 04) (Table 2). Pairwise comparisons of *ϕ*_ST_ ranged from 0.067 (Arb 97 and Arb 04) to 0.108 (Arb 04 and BB 04).

**Table 2.** Pairwise estimates of fixation (*F*_ST_, *ϕ*_ST_) and gene flow (Nm) between populations and generations. *F* statistics (*F*_ST_ in (a) and *ϕ*_ST_ in (b)) given below the diagonal and Nm given above. *F* statistics significantly different from zero (*P* < 0.05) are indicated with an asterisk.

(a) *F*_ST_	Arb 04	Arb 97	BB 04	BB 97	(b) *ϕ*_ST_	Arb 04	Arb 97	BB 04	BB 97
**Arb 04**		9.173	6.245	6.201	**Arb 04**		3.488	2.057	2.404
**Arb 97**	0.027*		7.400	8.292	**Arb 97**	0.067*		2.265	3.001
**BB 04**	0.038*	0.033*		28.961	**BB 04**	0.108*	0.099*		38.347
**BB 97**	0.039*	0.029*	0.009		**BB 97**	0.094*	0.077*	0.006	

In addition to pairwise comparisons, we used AMOVA to measure *F*_ST_ and *ϕ*_ST_ in both 1997 and 2004. We found that estimates of *F*_ST_ and *ϕ*_ST_ were low but significant and increased slightly from 1997 to 2004 (Table 3) **[see Supporting Information]**. AMOVA calculations of loci in HWE found low estimates of *F*_ST_ and *ϕ*_ST_ between populations in both years with no change in variance within populations across this time (92 % of allelic variance was found within populations for *F*_ST_ estimates in 1997 and 2004, and 86 % for ϕ_ST_ estimates in 1997 and 2004).

**Table 3.** Hierarchical AMOVA tables. The proportion of genetic variation is partitioned among populations, within populations and within individuals. Overall fixation, *F*_ST_, and corresponding Nm provided for both years (1997 in (a); 2004 in (b)). *F*_ST_ significantly different from zero (*P* < 0.05) are indicated with an asterisk.

	Source	df	SS	MS	Est. var.	%
(a) 1997						
	Among populations	1	16.602	16.602	0.143	5
	Among individuals	93	319.635	3.437	0.890	33
	Within individuals	95	157.500	1.658	1.658	62
	Total	189	493.737		2.691	100
	***F*_ST_**	0.053*				
	**Nm**	**4.448**				
(b) 2004						
	Among populations	1	34.977	34.977	0.195	7
	Among individuals	162	520.151	3.211	0.801	31
	Within individuals	164	264.000	1.610	1.610	62
	Total	327	819.128		2.605	100
	***F*_ST_**	0.075*				
	**Nm**	**3.088**				

**Figure 2.**
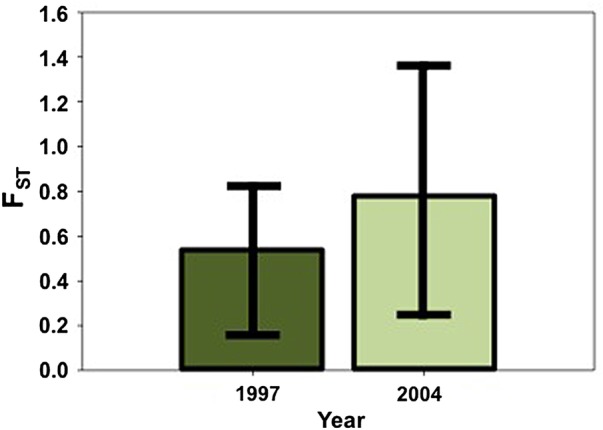
Population fixation (*F*_ST_) between 1997 and 2004. *F*_ST_ estimated with 1000 bootstrap replications. Error bars display 95 % CI.

To compare fixation between the two populations before and after the drought, we permuted *F*_ST_ (Fig. 2) using 1000 bootstrap replications. We estimated *F*_ST_ to be 0.053 (0.019–0.082; 95 % CI) between BB and Arb in 1997 and 0.076 (0.024–0.136) in 2004. As these estimates of *F*_ST_ do not overlap with zero, this suggests significant levels of differentiation between the populations in both years. However, the estimates of *F*_ST_ were not significantly different between these 2 years (Fig. 2), indicating a lack of change in fixation or gene flow between the generations. Analysis using loci in HWE similarly shows no significant change in *F*_ST_ between Arb and BB during this time.

**Figure 3.**
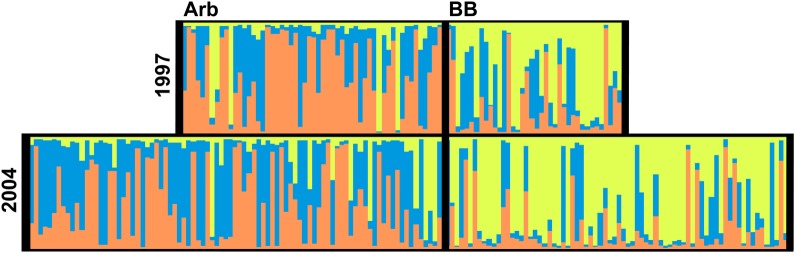
Genetic clustering assignment. Bayesian cluster analysis using allelic data from 10 microsatellite loci calculates three (*K* = 3) distinct genetic units (yellow, blue and orange). Assignment of these units is applied to each individual sampled in all four populations.

Bayesian analysis of this data suggests the existence of three distinct clusters (*K* = 3) in this system **[see Supporting Information]**. The greater proportion of individuals assigned to the genetic cluster represented by yellow in BB as compared with Arb indicates some genetic distinctiveness between these locations in both years. However, every individual sampled is estimated, in some proportion, to be assigned to each of these three clusters (yellow, orange and blue) (Fig. 3) suggesting low levels of structure, and consequently high levels of gene flow or genetic similarity in 1997 and 2004.

## Discussion

The results of this study show relatively high levels of inbreeding within the Arb and BB populations, and low fixation between these locations in 1997 and 2004. Despite a previous finding of reduced phenological, and potentially reproductive isolation between these populations following a 5-year drought, we did not find a significant change in gene flow or in genetic structuring across this time.

### Genetic diversity

Microsatellite data from these populations revealed high allelic diversity. Across all loci, we found 58 alleles, compared with 47 described in the literature **[see Supporting Information]** (Szewc-McFadden *et al*. 1996; Lowe *et al*. 2002, 2004; Suwabe *et al*. 2002). We found no significant shift in within population genetic parameters between 1997 and 2004 **[see Supporting Information]**. The presence of eight new PA in BB in 2004, as compared with 1997, may indicate an introduction of new alleles by mutation (Selkoe and Toonen 2006), as no nearby populations are known to exist to introduce new alleles via gene flow. Mutation rate is dependent on the effective population size and can vary due to environmental stresses, allowing for different trends in allelic diversity at each site (Hoffmann and Hercus 2000). However, the increase in PA in BB may also be due, fully or in part, to the relatively small sample size of BB 97 compared with BB 04 and a failure to detect low frequency alleles with our sampling effort. Thus, these populations appear to have high diversity at these loci, but there is little evidence to suggest changes in diversity over time.

### Intra-population genetic structuring

We found evidence for high levels of inbreeding in our populations despite the genetic self-incompatibility of *B. rapa* (Sobotka *et al*. 2000). This high level of inbreeding could be due to local seed and pollen dispersal causing mating between relatives, or to assortative mating. Some level of assortative mating was likely in this system due to the range of flowering times, as individuals may only cross with other individuals contemporaneously in flower (Weis and Kossler 2004; Franks and Weis 2009), and individuals with similar flowering times may be more likely related to one another than expected by chance. Levels of inbreeding did not change significantly between these years despite changes in the flowering phenology of each population (Franks and Weis 2009). As the drought resulted in an abbreviated growing season in 2004, this could allow for a greater proportion of individuals to be in flower at the same time thereby reducing the likelihood of potential crosses between closely related individuals. However, genetic similarity among all individuals may have allowed these populations to maintain high *F*_IS_ across these generations. Ultimately, this shift in phenology did not appear to influence the genetic structuring within each population.

### Fixation and gene flow over time

We found low levels of fixation, and correspondingly high levels of gene flow or shared common ancestry, between the two populations both before and after the drought. The lowest values of fixation were found between years within the same population, which is likely due to the 2004 generation maintaining much of the genetic character of its ancestral 1997 populations. However, we found very little deviation from these *F*_ST_ values between all other population comparisons, indicating maintenance of, and no large change in, population connectivity over these generations.

We calculated *F*_ST_ and *ϕ*_ST_ in both of these generations to investigate whether a change in gene flow occurred with the decreased phenological isolation between the populations. Although these parameters have been critiqued as estimates of genetic differentiation, particularly for microsatellite data (Whitlock and McCauley 1999; Jost 2008), the focus of our analysis is to determine if the populations became more genetically similar over time, as would occur if gene flow increased. Thus, examining these parameters and their change over time is appropriate for our goals. Both *F*_ST_ and *ϕ*_ST_ indicate similar patterns of fixation between the BB and Arb populations with lower, significant fixation occurring prior to the drought in 1997 and higher, significant fixation following the drought in 2004 (Table 3) **[see Supporting Information]**. These values of fixation are very low and represent minimal structure between the populations. The statistical significance of these measures suggests a deviation from no fixation, however the low estimates of *F*_ST_ may not represent biologically meaningful levels of structure. The 95 % bootstrapped CI indicate no significant change in *F*_ST_ or gene flow over time (Fig. 2). This result was not expected given the increased phenological opportunity for gene flow. However, in some systems, a decrease in phenological isolation may not cause changes in gene flow, particularly when minimal structure is present prior to the phenological change.

The data were also analysed using Bayesian statistics, which showed little genetic distinctiveness between these populations and generations. Three genetic clusters were recovered for this dataset, and all individuals in each population were assigned to these clusters to varying degrees (Fig. 3). This indicates a high degree of genetic relatedness of the BB and Arb sites across these years. Despite some variation across the two sites in terms of the relative proportions of each cluster, the assignment of all individuals sampled to each of the three clusters suggests that these populations had a recent shared ancestry. Furthermore, no major shift in proportion of these genetic units is evident across time (Fig. 3). Overall, this assessment of the data supports the results of *F*_ST_ analyses. The minimal observed change in structure and gene flow may have been due to an insignificant change in migration rate over time, or these results may have instead been impacted by the populations' genetic similarity or large effective population sizes (Neigel 2002).

### Evolutionary, environmental and community considerations

The lack of an expected change in gene flow over time could be due to several factors including the evolutionary history of these populations, seed banks, a low initial degree of genetic structure between the populations or a direct effect of the drought on the movement of pollen between populations. While research has been done to examine the phenological traits of these populations, little is known about their evolutionary history preceding the initial Franks *et al*. (2007) study, and pollination dynamics are not well understood. The low levels of fixation maintained between the two populations across seven generations (Table 3) **[see Supporting Information]** could have resulted if they had been very genetically similar across the data collection period. For example, if both populations had been established by individuals from the same source population, one population had been established from the other, or they were effectively a single population in the recent past, they would exhibit low levels of fixation. In any of these cases, fixation could have increased over time, given the 3 km separating these populations, allowing for divergence through local effects of genetic drift and mutation at neutral loci. However, it is possible that neither of these processes had an effect on fixation in this time, or that high levels of gene flow diminished a trend toward fixation.

Seed banks present in these populations might influence phenology and genetics. Both populations likely have persistent seed banks, as seed viability remains high over time, especially under the dry conditions that these populations experience outside of the growing season, and as persistent seed banks were found in the related species, *Brassica napus* (Simard *et al*. 2002). The presence of a seed bank would serve to hinder the evolutionary response to selection across this climatic shift, as genotypes adapted to previous conditions could emerge after conditions changed. However, evolutionary change in phenology was previously documented in these populations across these years (Franks *et al*. 2007) despite the likely presence of a seed bank. Nevertheless, a seed bank could have been a contributing factor to the lack of change in gene flow seen in these populations.

Additionally, given that little information exists regarding the pollinators of this system in these years, it is unknown whether they were able to easily traverse the 3 km distance separating the populations. Some sources define 3 km as the maximum distance that insects will forage (Rieger *et al*. 2002), while others believe that flowering plant populations within a range of 10–20 km can still exhibit substantial levels of gene flow, depending on the type of insect pollinator (dePamphilis and Wyatt 1989; Ellstrand 1992; Cresswell *et al*. 2000). Therefore, understanding whether the distance between these populations is a reasonable foraging range for this system's pollinators is necessary to properly interpret these results. The observed levels of fixation suggest very high levels of gene flow but this may instead be a consequence of historic patterns of population connectivity (Heywood 1991; Slatkin 1995; Whitlock and McCauley 1999).

The landscape surrounding these two populations is highly urbanized and developed (Fig. 1), but contains managed conservation areas surrounding the populations, including the UCI Arboretum grounds and the Upper Newport Bay Nature Preserve and Ecological Reserve. Although weedy populations of other wild and landscaped flowering plants are present in the area and could potentially serve as stepping stones in pollinators moving between the two study populations, the extent to which this occurs, and the effects of urbanization in this area on pollinator movement, is not known.

Another potential explanation for the absence of a significant change in gene flow with decreased phenological isolation is the effect of the drought, or other unknown changes, on pollinators. As *B. rapa* is primarily pollinated by insects, gene flow would require the foraging activity of these pollinators. As climate change is negatively impacting many systems across the globe (Hannah *et al*. 2002; Parmesan and Yohe 2003), the effect of this drought may have altered the population sizes of insects in the area or caused some modification of their behaviour that impacted their ability to transfer pollen across this range (Ollerton 1996; Rafferty and Ives 2011). Therefore, if the potential for increased gene flow existed in these populations over this drought period, negative impacts of the drought on pollinators could have had effects throughout the community, which may have minimized the level of gene flow achieved through this change (Ollerton 1996; Williams *et al*. 2008; Walther 2010; Rafferty and Ives 2011). As little is known about the evolutionary history of this system and about the community at the time of this drought, any variation in gene flow or genetic structuring cannot be directly attributed to a change in climate. However, an effect of the drought on the pollinators in this area would likely have consequences for gene flow (Cresswell *et al*. 2000).

## Conclusions

Despite the lack of a significant change in genetic structuring of these populations in response to a climate change event, this case highlights the importance of understanding the effects of environmental changes on gene flow between natural populations. Further research examining the genetic responses of natural populations to climate change across multiple systems will be useful in increasing our understanding of the evolutionary consequences of climate change (Franks and Hoffmann 2012) and establishing a baseline understanding with which to advise management decisions.

## Sources of Funding

Funding was provided by a Sigma Xi Grant-in-Aid-of-Research (G20101015154375) (USA) to R.S.W., a Faculty Research Grant from Fordham and a grant (DEB-1142784) from the National Science Foundation (USA) to S.J.F. and by the New York Botanical Garden.

## Contributions by the Authors

All authors took part in designing the experiment, discussing the results and composing and editing the manuscript. R.S.W. carried out the research, analysed the data and drafted the manuscript.

## Conflict of Interest Statement

None declared.

## Supporting Information

The following additional information is available in the online version of this article–

**Table S1.** ‘HWE estimates of *F*_IS_’ provides estimates of *F*_IS_, and corresponding 95 % CI, from 1000 bootstrap replicates using only loci found to be in HWE. Loci used are listed for each population and generation.

**Table S2.** ‘HWE estimates of *F*_ST_’ provides estimates of *F*_ST_, and corresponding 95 % CI (when available), from 1000 bootstrap replicates using only loci found to be in HWE. Loci used are listed for each generation.

**Table S3.** ‘Hierarchical AMOVA tables using loci in HWE’ (*F*_ST_) provides estimates of *F*_ST_ and the distribution of molecular variance for both generations, using only loci in HWE.

**Table S4.** ‘Hierarchical AMOVA tables using loci in HWE’ (*ϕ*_ST_) provides estimates of *ϕ*_ST_ and the distribution of molecular variance for both generations, using only loci in HWE.

**Table S5.** ‘Loci in HWE’ provides a list of the loci estimated to be in HWE for each population and generation.

**Table S6.** ‘Microsatellite loci characteristics’ lists allelic parameters calculated for each of the 10 loci used in this study.

**Table S7.** ‘Hierarchical AMOVA tables’ provides estimates of *ϕ*_ST_ and the distribution of molecular variance for both generations, using all 10 loci.

**Table S8.** ‘Within population parameters’ provides estimates of standard deviation for genetic parameters of each population and generation.

**Table S9.** ‘STRUCTURE likelihood outputs’ provides likelihood scores used to determine the optimal number of clusters comprising these populations.

**Table S10.** ‘Within population parameters over time’ provides the results of Student's *t*-test comparing within population genetic parameters across generations.

**Table S11.** ‘CI for within population fixation’ provides the 95 % CI corresponding to estimates of *F*_IS_ from 1000 bootstrap replicates, for each population and generation.

**Table S12.** ‘Estimates of *F*_IS_ across populations’ provides global estimates of *F*_IS_ for each generation, and across generations.

Additional Information
