# Supplementary material for: Analysis of population genetic structure and gene flow in an annual plant before and after a rapid evolutionary response to drought
Source: AoB Plants. 2015 Mar 27;7:plv026. doi: 10.1093/aobpla/plv026 (PMC4417203; doi:10.1093/aobpla/plv026)
Supplement: Additional Information [file supp_7_plv026_index.html]

Analysis of population genetic structure and gene flow in an annual plant before and after a rapid evolutionary response to drought — Additional Information 

# Analysis of population genetic structure and gene flow in an annual plant before and after a rapid evolutionary response to drought

## Additional Information

Additional Information

**Files in this Data Supplement:**

- Supplementary File 1 - docx file
- Supplementary File 2 - docx file
- Supplementary File 3 - docx file
- Supplementary File 4 - docx file
- Supplementary File 5 - docx file
- Supplementary File 6 - docx file
- Supplementary File 7 - docx file
- Supplementary File 8 - docx file
- Supplementary File 9 - docx file
- Supplementary File 10 - docx file
- Supplementary File 11 - docx file
- Supplementary File 12 - docx file
