# Supplementary material for: Analysis of population genetic structure and gene flow in an annual plant before and after a rapid evolutionary response to drought
Source: AoB Plants. 2015 Mar 27;7:plv026. doi: 10.1093/aobpla/plv026 (PMC4417203; doi:10.1093/aobpla/plv026)
Supplement: Additional Information [file supp_plv026_plv026supp_file1.docx]

**Supporting Information: HWE estimates of F_IS_.** Estimation of F_IS_ and 95% confidence intervals, from 1,000 bootstrap replications, for each population and year, using only loci in Hardy-Weinberg Equilibrium.

| **Population** | **Year** | **Mean** | **Lower Bound** | **Upper Bound** | **Loci Used** |
| --- | --- | --- | --- | --- | --- |
| **BB** | **1997** | 0.085 | 0.037 | 0.134 | Na10-A08, Ol10-D08, BRMS-037 |
| **BB** | **2004** | 0.071 | 0.004 | 0.156 | BN12A, Na10-D09, Ni4-A03, Ra2-E04 |
| **Arb** | **1997** | 0.065 | 0.023 | 0.104 | Na10-A08, Ni4-A03, Ra2-E04 |
| **Arb** | **2004** | 0.062 | -0.014 | 0.189 | BN12A, Na10-D09, Ol10-D08, Ra2-E04 |
