# Supplementary material for: Analysis of population genetic structure and gene flow in an annual plant before and after a rapid evolutionary response to drought
Source: AoB Plants. 2015 Mar 27;7:plv026. doi: 10.1093/aobpla/plv026 (PMC4417203; doi:10.1093/aobpla/plv026)
Supplement: Additional Information [file supp_plv026_plv026supp_file2.docx]

**Supporting Information: HWE estimates of F_ST_.** Estimation of F_ST_ and 95% confidence intervals, from 1,000 bootstrap replicates, between populations for each year, using only loci in Hardy-Weinberg Equilibrium.

| **Year** | **Mean** | **Lower Bound** | **Upper Bound** | **Loci Used** |
| --- | --- | --- | --- | --- |
| **1997** | 0.186 | - | - | Na10-A08 |
| **2004** | 0.132 | 0.057 | 0.299 | BN12A, Na10-D09, Ra2-E04 |
