# Supplementary material for: Analysis of population genetic structure and gene flow in an annual plant before and after a rapid evolutionary response to drought
Source: AoB Plants. 2015 Mar 27;7:plv026. doi: 10.1093/aobpla/plv026 (PMC4417203; doi:10.1093/aobpla/plv026)
Supplement: Additional Information [file supp_plv026_plv026supp_file3.docx]

**Supporting Information: Hierarchal AMOVA tables using loci in Hardy-Weinberg Equilibrium.** The proportion of genetic variation is partitioned among populations, within populations, and within individuals. Overall fixation, F_ST,_ and corresponding Nm provided for both years (1997 in a.; 2004 in b.). F_ST_ significantly different from zero at p < 0.05.

| **a. 1997** | | | | | | |
| --- | --- | --- | --- | --- | --- | --- |
| **Source** | **df** | **SS** | **MS** | **Est. Var.** | | **%** |
| **Among Pops** | 1 | 2.513 | 2.513 | 0.024 | | 8% |
| **Among Indiv** | 93 | 27.609 | 0.297 | 0.030 | | 10% |
| **Within Indiv** | 95 | 22.500 | 0.237 | 0.237 | | 81% |
| **Total** | 189 | 52.621 |  | 0.291 | | 100% |
| **F-Statistics** | **Value** | **P(rand >= data)** |  |  | |  |
| **Fst** | 0.083 | 0.002 |  |  | |  |
| **Nm** | 2.769 |  |  |  | |  |
| **b. 2004** | | | | | | |
| **Source** | **df** | **SS** | **MS** | | **Est. Var.** | **%** |
| **Among Pops** | 1 | 12.166 | 12.166 | | 0.069 | 8% |
| **Among Indiv** | 162 | 142.748 | 0.881 | | 0.059 | 7% |
| **Within Indiv** | 164 | 125.000 | 0.762 | | 0.762 | 86% |
| **Total** | 327 | 279.915 |  | | 0.891 | 100% |
| **F-Statistics** | **Value** | **P(rand >= data)** |  | |  |  |
| **Fst** | 0.078 | 0.001 |  | |  |  |
| **Nm** | 2.964 |  |  | |  |  |
