# Supplementary material for: Analysis of population genetic structure and gene flow in an annual plant before and after a rapid evolutionary response to drought
Source: AoB Plants. 2015 Mar 27;7:plv026. doi: 10.1093/aobpla/plv026 (PMC4417203; doi:10.1093/aobpla/plv026)
Supplement: Additional Information [file supp_plv026_plv026supp_file4.docx]

**Supporting Information: Hierarchal AMOVA tables using loci in Hardy-Weinberg Equilibrium.** The proportion of genetic variation is partitioned among populations, within populations, and within individuals. Overall fixation, φ_ST_, and corresponding Nm provided for both years (1997 in a.; 2004 in b.). φ_ST_ significantly different from zero at p < 0.05.

| **a. 1997** | | | | | |
| --- | --- | --- | --- | --- | --- |
| **Source** | **df** | **SS** | **MS** | **Est. Var.** | **%** |
| **Among Pops** | 1 | 5.025 | 5.025 | 0.096 | 14% |
| **Within Pops** | 93 | 55.217 | 0.594 | 0.594 | 86% |
| **Total** | 94 | 60.242 |  | 0.690 | 100% |
| **Stat** | **Value** | **P(rand >= data)** |  |  |  |
| **Φ_ST_** | 0.140 | 0.003 |  |  |  |
| **Nm** | 1.540 |  |  |  |  |
| **b. 2004** | | | | | |
| **Source** | **df** | **SS** | **MS** | **Est. Var.** | **%** |
| **Among Pops** | 1 | 24.332 | 24.332 | 0.277 | 14% |
| **Within Pops** | 162 | 285.497 | 1.762 | 1.762 | 86% |
| **Total** | 163 | 309.829 |  | 2.040 | 100% |
| **Stat** | **Value** | **P(rand >= data)** |  |  |  |
| **Φ_ST_** | 0.136 | 0.001 |  |  |  |
| **Nm** | 1.589 |  |  |  |  |
