# Supplementary material for: Analysis of population genetic structure and gene flow in an annual plant before and after a rapid evolutionary response to drought
Source: AoB Plants. 2015 Mar 27;7:plv026. doi: 10.1093/aobpla/plv026 (PMC4417203; doi:10.1093/aobpla/plv026)
Supplement: Additional Information [file supp_plv026_plv026supp_file5.docx]

**Supporting Information: Loci in HWE.** Loci within Hardy-Weinberg Equilibrium are denoted by an * for each population and year.

| **Population** | **Year** | **BN12A** | **Na10-A08** | **Na10-D09** | **Na10-G10** | **Ni4-A03** | **Ol10-D08** | **Ra2-E04** | **Ra2-E12** | **BRMS-040** | **BRMS-037** |  |  |  |  |  |
| --- | --- | --- | --- | --- | --- | --- | --- | --- | --- | --- | --- | --- | --- | --- | --- | --- |
| **BB** | **1997** |  | * |  |  |  | * |  |  |  | * |  |  |  |  |  |
| **BB** | **2004** | * |  | * |  | * |  | * |  |  |  |  |  |  |  |  |
| **Arb** | **1997** |  | * |  |  | * |  | * |  |  |  |  |  |  |  |  |
| **Arb** | **2004** | * |  | * |  |  | * | * |  |  |  |  |  |  |  |  |
