# Supplementary material for: Analysis of population genetic structure and gene flow in an annual plant before and after a rapid evolutionary response to drought
Source: AoB Plants. 2015 Mar 27;7:plv026. doi: 10.1093/aobpla/plv026 (PMC4417203; doi:10.1093/aobpla/plv026)
Supplement: Additional Information [file supp_plv026_plv026supp_file6.docx]

**Supporting Information: Microsatellite loci characteristics**: Locus identification, annealing temperatures (T_a_), number of alleles (A), expected heterozygosity (H_e_), observed heterozygosity (H_o_), and inbreeding coefficient (F_IS_). Standard error provided in parentheses.

Locus T_a_ (°C) A H_e_ H_o_ F_IS_

BN12A 51 2 0.457(0.021) 0.295(0.072) 0.354

Na10-A08 50 6 0.504(0.063) 0.346(0.082) 0.313

Na10-D09 58 5 0.616(0.029) 0.526(0.052) 0.146

Na10-G10 50 8 0.568(0.073) 0.191(0.017) 0.663

Ni4-A03 44 2 0.287(0.022) 0.191(0.034) 0.337

Ol10-D08 52 6 0.026(0.015) 0.011(0.007) 0.575

Ra2-E04 53 6 0.615(0.026) 0.553(0.034) 0.101

Ra2-E12 51 9 0.755(0.026) 0.273(0.026) 0.638

BRMS-040 49 6 0.497(0.014) 0.299(0.030) 0.398

BRMS-037 55 8 0.583(0.021) 0.598(0.033) -0.026

Mean 5.8 0.491(0.033) 0.328(0.031) 0.350(0.073)
