# Supplementary material for: Analysis of population genetic structure and gene flow in an annual plant before and after a rapid evolutionary response to drought
Source: AoB Plants. 2015 Mar 27;7:plv026. doi: 10.1093/aobpla/plv026 (PMC4417203; doi:10.1093/aobpla/plv026)
Supplement: Additional Information [file supp_plv026_plv026supp_file7.docx]

**Supplementary Information. Hierarchal AMOVA tables.** The proportion of genetic variation is partitioned among populations, within populations, and within individuals. Overall fixation, φ_ST_, and corresponding Nm provided for both years (1997 in a.; 2004 in b.). φ_ST_ significantly different from zero (p < 0.05) is indicated with an *.

| c. |  | | | | | |
| --- | --- | --- | --- | --- | --- | --- |
| 1997 | Source | df | SS | MS | Est. Var. | % |
|  | Among Pops | 1 | 33.204 | 33.204 | 0.573 | 8 |
|  | Within Pops | 93 | 639.270 | 6.874 | 6.874 | 92 |
|  | Total | 94 | 672.474 |  | 7.447 | 100 |
|  | φ_ST_ | 0.077* |  |  |  |  |
|  | Nm | 3.001 |  |  |  |  |
|  |  |  |  |  |  |  |
| d. |  | | | | | |
| 2004 | Source | df | SS | MS | Est. Var. | % |
|  | Among Pops | 1 | 69.954 | 69.954 | 0.780 | 11 |
|  | Within Pops | 162 | 1040.302 | 6.422 | 6.422 | 89 |
|  | Total | 163 | 1110.256 |  | 7.202 | 100 |
|  | φ_ST_ | 0.108* |  |  |  |  |
|  | Nm | 2.057 |  |  |  |  |
|  |  |  |  |  |  |  |
