# Supplementary material for: Analysis of population genetic structure and gene flow in an annual plant before and after a rapid evolutionary response to drought
Source: AoB Plants. 2015 Mar 27;7:plv026. doi: 10.1093/aobpla/plv026 (PMC4417203; doi:10.1093/aobpla/plv026)
Supplement: Additional Information [file supp_plv026_plv026supp_file8.docx]

**Supporting Information: Within population parameters.** Standard deviation (SD) is calculated from standard error (SE) for parameters listed in Table 1, to be used with Student’s t-test.

| **Pop** | **Year** | **N** | **A** | **SE** | **SD** | **H_e_** | **SE** | **SD** | **H_o_** | **SE** | **SD** |
| --- | --- | --- | --- | --- | --- | --- | --- | --- | --- | --- | --- |
| **BB** | **1997** | 39 | 3.7 | 0.633 | 3.953 | 0.517 | 0.071 | 0.443 | 0.332 | 0.057 | 0.356 |
| **BB** | **2004** | 75 | 5 | 0.715 | 6.192 | 0.503 | 0.066 | 0.572 | 0.349 | 0.065 | 0.563 |
| **Arb** | **1997** | 56 | 4.2 | 0.629 | 4.707 | 0.49 | 0.065 | 0.486 | 0.332 | 0.065 | 0.486 |
| **Arb** | **2004** | 89 | 4.1 | 0.737 | 6.953 | 0.453 | 0.068 | 0.642 | 0.301 | 0.066 | 0.623 |
