# Supplementary material for: Analysis of population genetic structure and gene flow in an annual plant before and after a rapid evolutionary response to drought
Source: AoB Plants. 2015 Mar 27;7:plv026. doi: 10.1093/aobpla/plv026 (PMC4417203; doi:10.1093/aobpla/plv026)
Supplement: Additional Information [file supp_plv026_plv026supp_file9.docx]

**Supporting Information: STRUCTURE likelihood outputs.** Analyses for K=1 to K=6, at four iterations. Delta K is estimated and used to determine optimal K, following Evanno et al (2005).

| **K** | **Reps** | **Mean LnP(K)** | **SD LnP(K)** | **Ln'(K)** | **\|Ln''(K)\|** | **Delta K** |
| --- | --- | --- | --- | --- | --- | --- |
| **1** | **4** | -4902.875 | 0.050 | NA | NA | NA |
| **2** | **4** | -4706.025 | 0.943 | 196.850 | 67.825 | 71.928 |
| **3** | **4** | -4577.000 | 0.726 | 129.025 | 113.825 | 156.845 |
| **4** | **4** | -4561.800 | 10.311 | 15.200 | 44.125 | 4.279 |
| **5** | **4** | -4502.475 | 10.406 | 59.325 | 9.725 | 0.935 |
| **6** | **4** | -4452.875 | 11.759 | 49.600 | NA | NA |
