# Supplementary material for: Analysis of population genetic structure and gene flow in an annual plant before and after a rapid evolutionary response to drought
Source: AoB Plants. 2015 Mar 27;7:plv026. doi: 10.1093/aobpla/plv026 (PMC4417203; doi:10.1093/aobpla/plv026)
Supplement: Additional Information [file supp_plv026_plv026supp_file10.docx]

**Supporting Information: Within population parameters over time.** Student’s t-test is used to determine significant differences of within population genetic parameters for populations (BB in a., and Arb in b.) between years.

| **a.** | **t** | **df-t** | **p-value** | **b.** | **t** | **df-t** | **p-value** |
| --- | --- | --- | --- | --- | --- | --- | --- |
| **A** | -1.361 | 106.7 | 0.087 | **A** | 0.103 | 141.7 | 0.541 |
| **H_e_** | 0.144 | 95.1 | 0.5574 | **H_e_** | 0.393 | 137.6 | 0.652 |
| **H_o_** | -0.197 | 107.1 | 0.422 | **H_o_** | 0.335 | 135.9 | 0.631 |
