# Supplementary material for: Analysis of population genetic structure and gene flow in an annual plant before and after a rapid evolutionary response to drought
Source: AoB Plants. 2015 Mar 27;7:plv026. doi: 10.1093/aobpla/plv026 (PMC4417203; doi:10.1093/aobpla/plv026)
Supplement: Additional Information [file supp_plv026_plv026supp_file11.docx]

**Supporting Information: Confidence intervals for within population fixation.** 95% confidence intervals about mean F_IS_ within each population and year is estimated by 1,000 bootstrap replications.

| **Population** | **Year** | **Mean** | **Lower Bound** | **Upper Bound** |
| --- | --- | --- | --- | --- |
| **BB** | **1997** | 0.370 | 0.212 | 0.524 |
| **BB** | **2004** | 0.313 | 0.081 | 0.497 |
| **Arb** | **1997** | 0.330 | 0.154 | 0.513 |
| **Arb** | **2004** | 0.340 | 0.123 | 0.529 |
