# Supplementary material for: Analysis of population genetic structure and gene flow in an annual plant before and after a rapid evolutionary response to drought
Source: AoB Plants. 2015 Mar 27;7:plv026. doi: 10.1093/aobpla/plv026 (PMC4417203; doi:10.1093/aobpla/plv026)
Supplement: Additional Information [file supp_plv026_plv026supp_file12.docx]

**Supporting Information: Estimates of F_IS_ across populations.** Mean F_IS_, and corresponding standard deviation (SD), estimated across both populations, Arboretum and Back Bay, for both years, and for populations between years (given in ‘Total’).

| **Year** | **Mean** | **SD** |
| --- | --- | --- |
| **1997** | 0.366 | 0.077 |
| **2004** | 0.055 | 0.026 |
| **Total** | 0.350 | 0.073 |
